# Dual-Responsive Amphiphilic P(DMAEMA-co-LMA-co-OEGMA) Terpolymer Nano-Assemblies in Aqueous Media

**DOI:** 10.3390/nano12213791

**Published:** 2022-10-27

**Authors:** Maria Tomara, Dimitrios Selianitis, Stergios Pispas

**Affiliations:** Theoretical and Physical Chemistry Institute, National Hellenic Research Foundation, 48 Vassileos Constantinou Avenue, 11635 Athens, Greece

**Keywords:** amphiphilic statistical terpolymers, RAFT polymerization, dual-responsive copolymers, drug encapsulation, FBS, bio-imaging

## Abstract

This work reports on the synthesis and self-assembly of a novel series of dual-responsive poly[2-(dimethylamino)ethylmethacrylate-co-laurylmethacrylate-co-(oligoethyleneglycol)methacrylate], P(DMAEMA-co-LMA-co-OEGMA)statistical terpolymers in aqueous solutions. Five P(DMAEMA-co-LMA-co-OEGMA) amphiphilic terpolymers, having different content of the three monomers, were synthesized via reversible addition-fragmentation chain transfer (RAFT) polymerization. The success of the synthesis was confirmed by the molecular characterization of the terpolymers via size exclusion chromatography (SEC) for the determination of molecular weights and the molecular weight distributions. By using nuclear magnetic resonance (^1^H-NMR) and Fourier-transform infrared (FTIR) spectroscopy, it was possible to determine the exact composition of the terpolymers. Dynamic light scattering (DLS) and fluorescence spectroscopy (FS) indicated the formation of P(DMAEMA-co-LMA-co-OEGMA) unimolecular or multichain aggregates in aqueous solutions, as a response to pH, temperature and ionic strength changes, with their dimensions being largely affected. The amphiphilic terpolymers were able to encapsulate the hydrophobic drug curcumin (CUR) and demonstrate stability to fetal bovine serum (FBS) solutions. These terpolymer aggregates were studied by DLS, FS and UV-Vis, and it was found that they may have been used as potential nanocarriers for drug delivery and bio-imaging applications.

## 1. Introduction

Copolymers that consist of monomeric units that dissolve into two types of solvents are called amphiphilic (amphi: of both species, philic: having an affinity for). The scientific community has been intensely interested in amphiphilic copolymers composed of hydrophilic and hydrophobic monomers, due to their excellent properties, both in solid state and in aqueous media. The differing solubility and mutual incompatibility of the components results in the formation of interesting nanostructured morphologies, with the most characteristic one being their ability to self-organize in solution, which leads to the formation of organized structures, usually micelles, depending on the solvent in which they are dissolved and their composition. The colloidal properties of these systems are due to micellization [1,2,3]. Amphiphilic block copolymers (AmBCs) have been extensively studied and used in pharmaceutical applications, from drug delivery studies to gene therapy [4].During micellization, the hydrophobic cores act as “containers” for the entrapment of hydrophobic drugs or imaging agents. Until recently, much emphasis has been placed on understanding the self-assembly of AmBCs, due to their unique and exceptional behavior of changing conformation, depending on the environment in which they are located, for example, when they are inserted into a selective solvent. However, their synthetic process can be time consuming, as it involves sequential, controlled polymerization or post-polymerization treatments [2].

Statistical (or random) copolymers are an asset at an industrial level, compared to block copolymers, as their synthetic process is much easier, since it can be achieved in one stage by copolymerization of two or more monomers. Therefore, it is worth studying their properties in terms of self-assembly [5,6,7]. When water is used as a solvent, their self-assembly is highly dependent on the hydrophilic/hydrophobic component ratio, leading to a great variation of morphologies. These morphologies are the result not only of the ratio of hydrophilic to hydrophobic segments but also of the length of the chains as well. The self-assembly of these polymers is based on intramolecular change of their chain conformation (intramolecular self-folding), creating monomeric micelles (unimer micelles), while in AmBCsmultichain, core–shell micelles are formed. More specifically, when the content in hydrophobic segments is considerably lower than the hydrophilic, the statistical copolymers fold intramolecularly, forming unimolecular micelles/nanoparticles with a hydrophobic core. On the contrary, by increasing the content of hydrophobic segments, intermolecular self-assembly is favored and, in this way, multichain aggregates are formed. As the hydrophobic content increases in a statistical copolymer (with degree of polymerization, DP, lower than composition-dependent threshold degree of polymerization, DP_th_), the size increases as well, as the system self-organizes intermolecularly. This is due to the DP threshold a specific degree of polymerization that depends on the composition of each statistical copolymer system. Molecular weight strongly correlates to the composition of the copolymers, and, as a result, statistical copolymers with wide distributions of molecular weight self-organize into nano-aggregates due to the simultaneous intramolecular folding of polymer chains with DP lower than the DP_th_ [8,9].

In addition to the amphiphilic character of copolymer systems, it is possible to make them respond to external stimuliby using appropriate sets of monomers. Such stimuli can be temperature, pH and ionic strength [10,11,12]. The main advantage of a stimuli-responsive polymer system for a drug-targeting strategy is the stimuli response, unique to a disease’s pathology, allowing the drug or gene delivery system to respond specifically to the specific pathological stimuli. Such “smart” vectors, based on amphiphilic copolymers, can be considered as active targeting systems, as they incorporate drugs that are released by the vectors in response to external or environmental changes.

Polymers containing alkaline or acidic functional groups respond to changes of pH [10,13,14]. Unlike acidic, “smart” polymers ionized at high pH, basic polymers are ionized at lower pH values. For example, poly(acrylic acid) (PAA) ionizes and dissolves at high pH, while poly(diethylamino ethyl methacrylate) (PDEAEMA) ionizes and dissolves at low pH, similar to poly(dimethylamino ethyl methacrylate) (PDMAEMA).Another parameter that is easier to control is temperature. Thermo-responsive polymers are capable of undergoing a phase transition at a certain temperature, thus, leading to changes in conformation, solubility and hydrophilic/hydrophobic equilibrium [4,11,15,16,17]. One of the most important thermo-responsive copolymers is poly(N-isopropylacrylamide) (PNIPAM) with a lower critical solution temperature (LCST) equal to 32 °C, close to normal human temperature of 37 °C [18].

RAFT polymerization is one of the most flexible and powerful polymerization techniques for obtaining copolymers of various architectures. It is a flexible method for synthesizing “living” polymers, giving control over polymer molecular weight, molecular weight distribution, composition and architecture. It is suitable for most monomers that are polymerized by radical polymerization and is tolerant to a fairly wide range of reaction conditions. Compared to other polymerization techniques, such as anionic polymerization, it can be performed even at room temperature, with water as a solvent and without the need to use protecting groups for the monomers utilized. Additionally, it enables the synthesis of block copolymers, star, graft and cyclic polymers, functional polymers, etc. In addition, the integration of functional monomers and the selection of the appropriate chain transfer agent (CTA) allows easy modification of the side or end groups after the polymerization is completed [19,20,21,22].

The goal of the present study is to synthesize, molecularly characterize and study the nanoscale assembly in aqueous solutions of a series of novel multi-responsive amphiphilic statistical terpolymers of the type P(DMAEMA-co-LMA-co-OEGMA), where DMAEMA = dimethyamino ethyl methacrylate, LMA = lauryl methacrylate and OEGMA = oligo ethylene glycol methacrylate. Furthermore, we investigate the abilities of these copolymers to act as drug nanocarriers. DMAEMA was used as the dual-responsive part of the macromolecule to pH and temperature changes. It is an extensively studied monomer, giving a weak polyelectrolyte of pK_a_ = 7.4 after polymerization and, additionally, it demonstrates an LCST in water in the range 32–50 °C, depending on the PDMAEMA molecular weight [23].Thus, pH and temperature variations of aqueous solutions could lead to a corresponding dependence of the properties of the terpolymer systems in terms of self-assembly. PLMA is a hydrophobic polymer, since there is a long hydrocarbon side chain on each segment and it presents a low Tg value, is not stimuli-responsive, but it contributes to the self-assembly of the macromolecules containing it in aqueous media due to hydrophobic interactions with the solvent [24]. Last but not least, POEGMA is the hydrophilic component;it is also not responsive. However, it demonstrates an important role in self-assembly, as it is usually the outer part of the aggregates; encloses the hydrophobic part; stabilizes it; and prevents it from precipitation [25].Finally, it is worth mentioning the important role of aggregate architecture. These are systems with random arrangement of monomeric units along the polymer chain, so the final morphology of the aggregates in the solution may not be entirely defined, as in the case of the block copolymers.

Skandalis et al. reported the synthesis and self-assembly behavior of linear PDMAEMA-b-PLMA-b-POEGMA triblock terpolymers and their quaternized derivatives QPDMAEMA-b-PLMA-b-POEGMA) [21]. Kafetzi et al. reported the synthesis and self-assembly in aqueous media of P(DMAEMA-co-LMA)-b-POEGMA random-block copolymers and of their cationic analogues P(QDMAEMA-co-LMA)-b-POEGMA [26].The synthesis of similar terpolymers with statistical arrangement of the same monomers along the polymeric chain is a piece of work reported for the first time; their properties in aqueous solutions, as a function of pH and temperature changes, as well as their behavior as drug carriers, are thoroughly studied in this work.

## 2. Materials and Methods

### 2.1. Materials

The monomers used were 2-(dimethylamino) ethyl methacrylate (DMAEMA) (98%), lauryl methacrylate (LMA) (96%) and (oligo ethylene glycol) methacrylate (OEGMA, average M_n_ = 475) from Sigma-Aldrich (St. Louis, MO, USA). They were purified by passing through an inhibitor removing mini column. Resins used were 311,340 and 311,332 from Sigma-Aldrich. The 2,2-azobis(isobutyronitrile) (AIBN) was the radical initiator utilized after recrystallization from methanol. The 1,4-dioxane (99.8% pure), from Aldrich, was dried over molecular sieves before use. Tetrahydrofuran (THF, 99.9% pure), 4-cyano-4-(phenylcarbonothioylthio) pentanoic acid (CPAD), fetal bovine serum (FBS) and other reagents from Aldrich were used as received.

### 2.2. P(DMAEMA-co-LMA-co-OEGMA) Synthesis

The synthesis of the five P(DMAEMA-co-LMA-co-OEGMA) terpolymers was accomplished in a one-step RAFT polymerization. After purification, the three monomers were placed in a 25 mL round-bottom flask dissolved in 8 mL of 1,4-dioxane, where both AIBN and the CTA in a ratio of 1:5 (mol) were placed previously. The exact quantities utilized are presented in Table S1. A Teflon-coated magnetic stir bar was added and the flask was fitted with a rubber septum. The final mixture was degassed for 20 min nitrogen gas bubbling and was subsequently placed in an oil bath at 70 °C, for 24 h. After this period, the reaction solution was cooled at −20 °C and exposed to air in order to ensure termination of the polymerization reaction. Finally, the P(DMAEMA-co-LMA-co-OEGMA) terpolymers were precipitated in an excess of n-hexane. P(DMAEMA-co-LMA-co-OEGMA)-4, which had the highest hydrophobic content (≃50% LMA), was an exception and the precipitation was not successful. The n-hexane/1,4-dioxane mixture was then evaporated using a rotary evaporator. The final product was collected and placed in a vacuum oven for 48 h to dry like the rest of the terpolymers.

### 2.3. Self-Assembly of P(DMAEMA-co-LMA-co-OEGMA) in Aqueous Media

The amphiphilic terpolymers were studied for their ability to self-assemble in aqueous solutions. Two protocols were followed. In the first, the polymer was directly dissolved in deionized water at pH = 7. Deionized water, about 10 mL, was added to a vial together with a weighted amount of polymer, about 10 mg (c = 1 × 10^−3^ g/mL). Stirring followed until the polymer was completely dissolved at ambient temperature. The solutions were left overnight for complete dissolution and equilibration. For the TER-4 terpolymer, it was necessary to use ultrasound for about 5 min in order to achieve complete dissolution of the material. Due to the thermo-responsive and pH-responsive nature of the DMAEMA units, the terpolymer solutions were adjusted afterwards to the appropriate pH using a proper quantity of 1 M HCl and 0.1 M NaOH, accordingly. In addition, each polymer was dissolved in phosphate-buffered saline (PBS) (pH = 7.4, NaCl 0.15 M), which mimics the conditions of salinity and acidity in blood. In the second protocol, thin-film hydration was performed. Initially, the terpolymers were dissolved in a non-selective solvent for all components (THF), and then the THF was evaporated using a rotary evaporator. Finally, hydration of the formed film was performed in order to achieve self-assembly of the chains in the aqueous environment. For fluorescence spectroscopy (FS) studies, pyrene was used as a tracer and a 1 mM stock solution in acetone was added in appropriate quantity to each P(DMAEMA-co-LMA-co-OEGMA) terpolymer solution of different pH. The solutions with pyrene were left overnight in order to reach equilibrium (final c_pyrene_ = 10^−7^ M). For dynamic light scattering studies, hydrophilic PVDF 0.45 μm filters were used to filter the solutions before measurements.

### 2.4. Ionic Strength Study

Another typical property of responsive polymers containing ionic groups is their ionic strength response. Changes in ionic strength, combined with changes in pH, can cause changes in the polymer nanoparticle size, solubility, and fluorescence quenching kinetics of bound/encapsulated chromophores.

The ionic strength effect study was performed by adding NaCl 1 M. Nine additions resulting in increasingly different salt concentrations were made to the aqueous solutions and changes in the scattering intensity and R_h_ was measured via dynamic light scattering (DLS). The measurements were recorded at 90° angle.

### 2.5. Curcumin Encapsulation into P(DMAEMA-co-LMA-co-OEGMA) Aggregates

Two different protocols were used to encapsulate curcumin in the polymeric aggregates. In the first protocol, two different solutions were prepared, one of the polymer in THF (for each terpolymer) and one of curcumin in THF. Specifically, 10 mg of each terpolymer were added to vials with 1 mL THF in each, until dissolved. In a second vial a proper amount of curcumin was added and dissolved in 1 mL THF. The solutions were left for about 24 h in order to dissolve molecularly. Afterwards, each terpolymer solution was mixed with the proper amount of curcumin solution and injected into new vials, containing 10 mL PBS under vigorous stirring. By heating the mixed solution the organic solvent was evaporated. In the second protocol, the thin-film hydration method (TFHM) was utilized. The terpolymer–curcumin sample (dissolved in THF) was placed in a round-bottom flask (25 mL), followed by evaporation of the solvent in a rotary evaporator. After complete evaporation, a thin film was formed around the inner walls of the flask, which was then hydrated with a small amount (10 mL) of PBS. Gentle agitation followed to redissolve the film. Calculations of the amount of curcumin were performed, taking into account the content of the hydrophobic component (wt% LMA) in each terpolymer, as well as the desired content of curcumin encapsulated in the nanoparticles. The mixed nanoparticles were studied by DLS, FS and UV-Vis, the 1st and the 10th day of their preparation. The absorption vs. curcumin concentration reference curve was constructed measuring at λ_max_ = 420 nm. Based on the calibration curve and the absorbance value of each sample, the amount of curcumin encapsulated by each terpolymer was calculated.

### 2.6. FBS Interaction Study

Curcumin encapsulating polymeric systems were interacted with fetal bovine serum (FBS) protein solutions in order to have an initial check on their blood compatibility. On the first day, 3 mg of polymer-curcumin was dissolved in 1 mL of PBS following the thin-film protocol. The next day, 4 mL of the FBS solution were filtered and used. Two protocols were followed in order to follow the effect of polymer concentration. In the first, 50 μL of the polymer–curcumin solution were added to 3 mL of FBS:PBS solution (in a FBS:PBS ratio of 1:9, 10% *v*/*v*–90% *v*/*v*) and 50 μL of the same solution were added to 3 mL of FBS:PBS solution (in a FBS:PBS ratio of 1:1, 50% *v*/*v*–50% *v*/*v*). In the second protocol, 100 μL of the polymer–curcumin sample were added to 3 mL of 1:9 FBS:PBS solution, and then the same amount of the mixed system was added to 3 mL of 1:1 FBS:PBS solution. The prepared samples were measured after 3 h by DLS. The results were compared with the corresponding FBS-filtered solution.

### 2.7. Characterization Methods

#### 2.7.1. Size Exclusion Chromatography (SEC)

**Figure 1:**
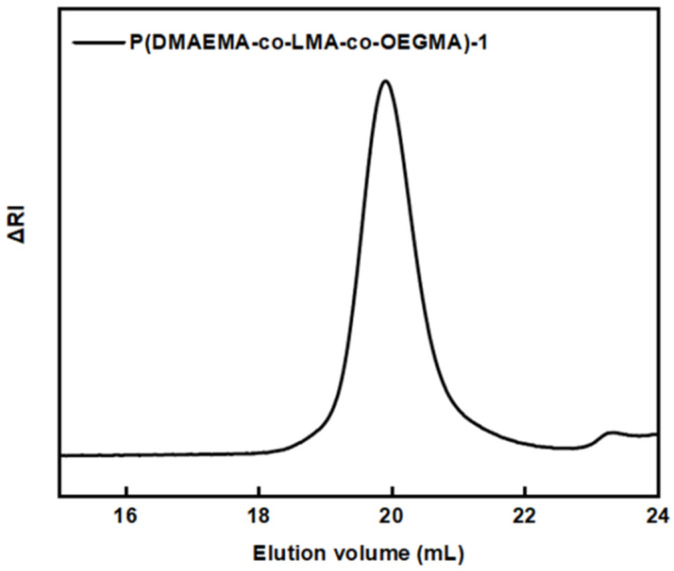
SEC chromatogram for TER-1 terpolymer.

The molecular weights and molecular weight distributions of the synthesized polymers were determined by size exclusion chromatography. A Waters SEC set-up was used, consisting of an isocratic Waters 1515 pump, a set of three μ-Styragel mixed composition separation columns (10^2^–10^6^ Å pore range), a Waters 2414 refractive index detector (at 40 °C) and breeze-controlled software. Tetrahydrofuran was used as the solvent, which contained 5% *v*/*v* triethylamine, at a flow rate of 1 mL/min at 30 °C. The instrument was calibrated with standard polystyrene samples with narrow molecular weight distributions and average molecular weights in the range of 2500 to 123,000 g/mol. A typical chromatogram is shown below in Figure 1.

#### 2.7.2. ^1^H-NMR Spectroscopy

^1^H-NMR spectra were obtained using a Bruker AC 600 FT-NMR spectrometer with tetramethylsilane (TMS) as the reference. The preparation of solutions for the ^1^H-NMR measurements involved dissolving 10 mg of the polymer 0.7 mL deuterated chloroform. The spectral analysis was performed using MestReNova software from MestReLabs (Santiago de Compostela, Spain).

**Figure 2:**
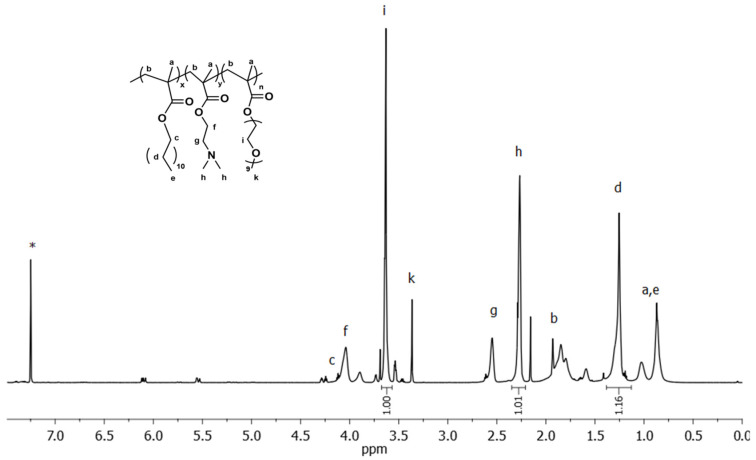
^1^H-NMR spectrum for TER-5 terpolymer (* corresponds to CDCl_3_ solvent and letters to the corresponding hydrogens shown on the chemical structure).

^1^H-NMR peaks of P(DMAEMA-co-LMA-co-OEGMA) (Figure 2, 300 MHz, CDCl_3_, ppm): 0.83–1.00 (3H, C*H_3_*C–, 3H, C*H_3_*(CH_2_)_10_–), 1.25 (20H, –(C*H_2_*)_10_–), 1.75 (2H, –C*H_2_*CCH_3_–), 2.27 (6H, (C*H_3_*)_2_N–), 2.55 (2H, –NC*H_2_*–), 3.30 (3H, C*H_3_*–(OCH_2_CH_2_)_9_–), 3.36 (36H, –(OC*H_2_*C*H_2_*)_9_–), 4.10–4.25 (2H, –COOC*H_2_*–, 2H, –(CH_2_)_10_C*H_2_*COO–).

#### 2.7.3. Dynamic Light Scattering (DLS)

Dynamic light scattering measurements were performed utilizing a ALV/CGS-3 Compact Goniometer System (ALV GmbH, Langen, Germany), with a JDS Uniphase 22 mW He-Ne laser at 632.8 nm, connected to a 288-channel ALV-5000 digital coupler/EPP multi-tau correlator and a light scattering module ALV/LSE-5003 for goniometer arm control. The intensity of the scattered light and the correlation functions were recorded five times and analyzed by the methods of cumulants and CONTIN. The latter provides the distributions for the apparent hydrodynamic radius using the inverse Laplace transform of the autocorrelation function and the Stokes–Einstein equation. All solutions were filtered through 0.45 μm porous hydrophilic PVDF filters before measurement. Measurements vs. temperature were carried out in the range of 25 to 55 °C, at 5 °C steps, allowing for 15 min equilibration between temperatures. The size data presented below are from measurements at 90° measuring angle.

#### 2.7.4. Fluorescence Spectroscopy (FS)

The spectra were recorded with a NanoLog Fluorimeter (Horiba Jobin Yvon, Kyoto, Japan), using a laser diode as the excitation source (NanoLED, 440 nm, pulse width 100 ps) and a UV TBX-PMT series detector (250–850 nm) by Horiba Jobin Yvon. The solutions utilized were prepared in a concentration range of 10^−3^–10^−8^ g/mL. Pyrene solution in acetone at a ratio of 1 μL/mL was added to each vial. The samples were kept at rest for 24 h to ensure encapsulation of pyrene into the hydrophobic domains of the polymer aggregates. The I_1_/I_3_ ratio vs. concentration was then measured. The excitation wavelength used for the measurements was 335 nm. Emission spectra were recorded in the spectral range 355–640 nm. For the fluorescence measurements on CUR-loaded terpolymers, the same instrument was utilized. Curcumin excitation wavelength was set at 405 nm in this case.

#### 2.7.5. UV-Vis Spectroscopy

UV-Vis spectra were recorded using a Perkin-Elmer (Lamda 19, Waltham, MA, USA) UV-Vis spectrophotometer. Quartz cells were used, in which 3 mL of the solution to be measured was placed.

#### 2.7.6. Electrophoretic Light Scattering (ζ-Potential)

ζ-potential measurements were implemented using a Malvern system (Nano Zeta Sizer, Malvern Instruments Ltd., Malvern, UK) equipped with a He-Ne 4 mW laser at λ = 633 nm wavelength. The scattered radiation is measured at an angle of 173°. Electrokinetic measurements to determine the mobility and ζ-potential values of colloids were performed using the LDV (laser Doppler velocimetry) technique and the Smoluchowski approach for analysis. The reported ζ-potential values are the average of 100 measurements.

## 3. Results and Discussion

### 3.1. Synthesis and Molecular Characterization

**Scheme 1:**
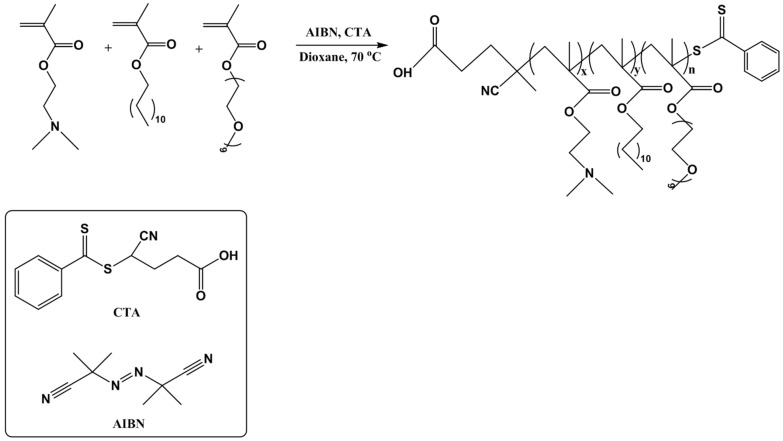
Synthesis of P(DMAEMA-co-LMA-co-OEGMA) terpolymers via RAFT polymerization.

The synthesis of all five amphiphilic statistical terpolymers of the type P(DMAEMA-co-LMA-co-OEGMA) was performed by RAFT polymerization, as described in Scheme 1 below. As a CTA reagent, CPAD was selected for the polymerizations, as it is known from literature for its effectiveness in RAFT polymerization of methacrylic monomers [19].AIBN was utilized as a radical initiator and 1,4-dioxane as the polymerization solvent. The five terpolymers have different compositions by weight for each monomer. Based on the results obtained by SEC, the polymerization scheme has been proven successful. A typical chromatogram of a P(DMAEMA-co-LMA-co-OEGMA) terpolymer is shown in Figure 1. Analysis of the five chromatograms indicated the successful control of the molecular weight of the terpolymers, and the values of the molecular weight distributions were quite small (Đ < 1.2), as required by the theoretical background of the RAFT polymerization technique. It should be noted that molecular weights determined by the SEC are the apparent ones. Table 1 presents the molecular characteristics of all five terpolymers synthesized.

The identification of the expected chemical structure and the determination of the composition of the terpolymers were performed by ^1^H-NMR spectroscopy. A characteristic spectrum is presented below (Figure 2), along with the evaluation of characteristic peaks. The intensity and width of the peaks were different on each spectrum, depending on the composition of the terpolymers.

For the calculation of the composition of each system, the signals of –CH_2_– protons corresponding to LMA appearing at 1.25 ppm, the –CH_2_– protons of OEGMA appearing at 3.63 ppm and the –CH_3_ protons of the two methyl groups on the amino side group of DMAEMA appearing at 2.27 ppm were utilized [22,27].

### 3.2. Self-Assembly in Aqueous Media

The preparation of the terpolymer aqueous solutions was performed, as mentioned in the experimental section. The protocol for the formation of aggregates was common to all five terpolymers. By fluorescence spectroscopy it was investigated whether the aqueous solutions formed aggregates, calculating the critical aggregate formation concentration (CAC). This study was performed to investigate how the statistical arrangement of the monomers affects the formation of aggregates. The measurements were performed by trapping pyrene in the hydrophobic domains of the terpolymer aggregates, as it is a hydrophobic fluorescent compound with low water solubility. Its fluorescence is sensitive to changes in the polarity of its immediate environment. An increment in the hydrophobic component of the terpolymers also means a higher percentage of pyrene entrapped in the aggregates and, therefore, a lower I_1_/I_3_ ratio. The terpolymer solutions were prepared in concentrations, ranging from 10^−8^–10^−3^ g/mL. The acquired values for each terpolymer at different pH rates are shown in Table S2, along with the CAC values for each polymer system.

**Figure 3:**
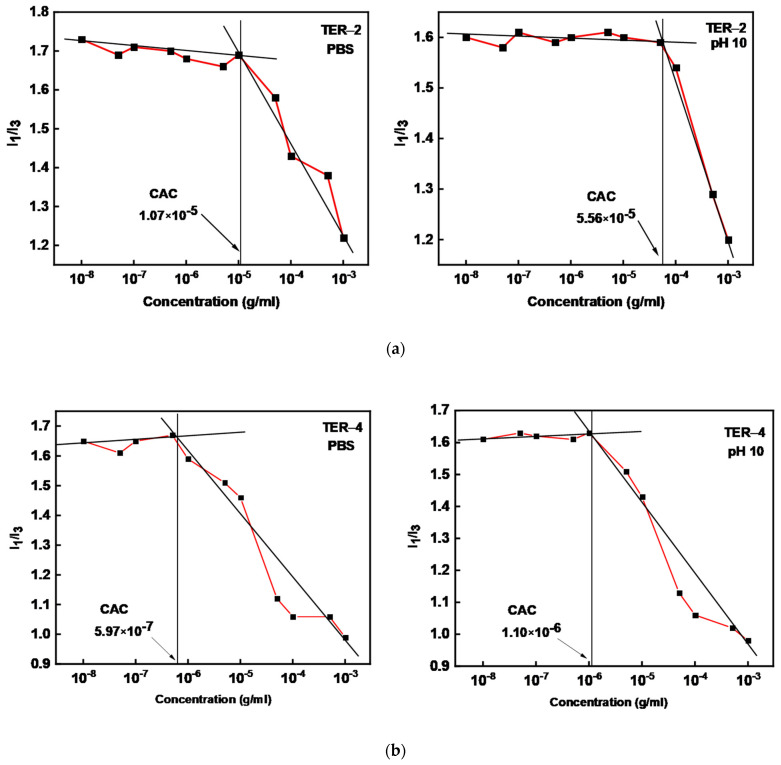
(**a**) CAC determination for TER-2 terpolymer; (**b**) TER-4 terpolymer in PBS (**left**) and pH = 10 (**right**), respectively. Red lines: are a guide to the eye, connecting all experimental points; Black lines: show how CAC was determined by the intersection point.

I_1_/I_3_ values vs. copolymer concentration from pyrene fluorescence spectra at pH = 7.4 (PBS) and pH = 10 are illustrated in Figure 3. At low concentrations, the plateaus are clearly observed, where no aggregates have been formed. Additionally, the transition at intermediate concentrations is observed and copolymer aggregates are mainly formed at higher concentrations. From the first turning point of the two interpolated straight lines, as shown, the CAC is determined. It is obvious that the CAC values for TER-2 copolymer are higher than those for TER-4, which is due to the lower content of the hydrophobic LMA component. The CAC values are given in Table S2. TER-4 copolymer typically has the lowest CAC value, due to its highest content in LMA. Corresponding reductions are observed in the ratio I_1_/I_3_, depending on the LMA content. In addition to the composition in LMA, the pH-responsive DMAEMA response is important, as these type of segments are fully deprotonated at pH = 10, leading to increased entrapment of pyrene and the formation of aggregates. At pH = 7, DMAEMA segments are partly deprotonated and there is a transition to a fairly hydrophilic state, whereas at pH = 3, DMAEMA segments are completely protonated and even more hydrophilic, giving rise to a higher I_1_/I_3_ ratio. The effect of temperature changes is also shown in Table S2.

**Figure 4:**
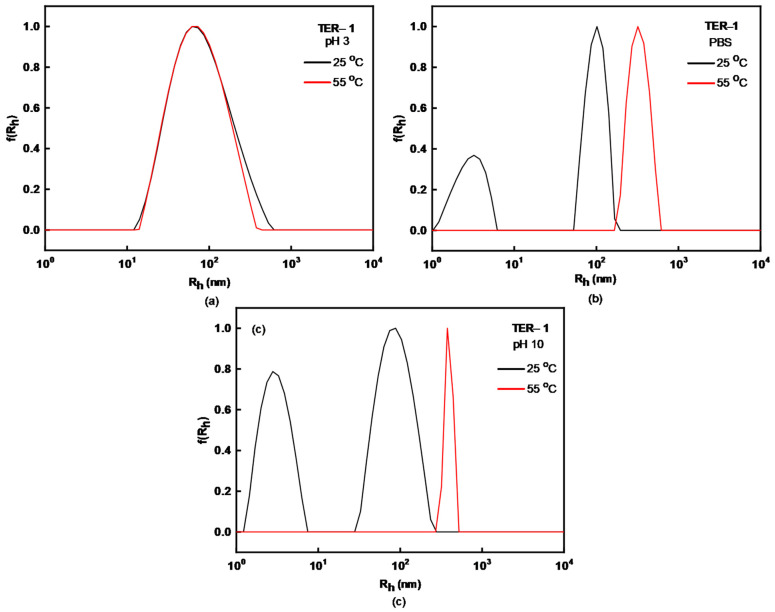
CONTIN size distributions for TER-1 terpolymer at (**a**) pH = 3, (**b**) PBS and (**c**) pH = 10, as a function of temperature.

In order to determine the hydrodynamic radius (R_h_) and the size polydispersity index (PDI) of the polymeric aggregates, dynamic light scattering (DLS) was performed. Scattering light intensity is strongly related to the mass of the formed nanoparticles. In Figure 4, the size distributions of the aggregates for TER-1 in three different pH values, at 25 °C and 55 °C, are presented. At pH = 3 the polymeric chains are fully protonated and at both 25 °C and 55 °C, a relatively wide monomodal size distribution is observed, indicating one population of aggregates in the solution. The large apparent size of the aggregates may be consistent with the existence of electrostatic interactions between charged polymer chains. An increase in temperature does not seem to affect the R_h_ distribution. The self-assembly of the nanoparticles in PBS (pH = 7.4) solutions at 25 °C seems to lead to the formation of two populations of different sizes. Part of the polymer system seems to be in the form of free chains, while the rest is in the form of aggregates formed due to the partial deprotonation of PDMAEMA, which results in lower solubility/higher hydrophobic interactions of the polymer chains. At 55 °C, the polymer forms a single population of aggregates having larger dimensions with relatively lower size distribution. This is due to further aggregation of the existing polymer nanoparticles/chains, which is a result of the reduced hydrophilicity of PDMAEMA parts by the increasing temperature. At pH = 10 two populations are observed again at 25 °C, with a transition to one larger population at 55 °C. Obviously, the increase in temperature here also favors the aggregation of the particles towards larger aggregates. The first (low R_h_) peak in PBS solutions at 25 °C remains at the same position and increases in intensity when changing the pH to 10, probably due to the aggregation of free chains that may have remained, giving aggregates of similar size but larger mass. The environment is now alkaline, with PDMAEMA being completely deprotonated and, therefore, there is an enhancement of hydrophobic interactions, resulting in large size aggregates at 55 °C (and pH 10).

**Figure 5:**
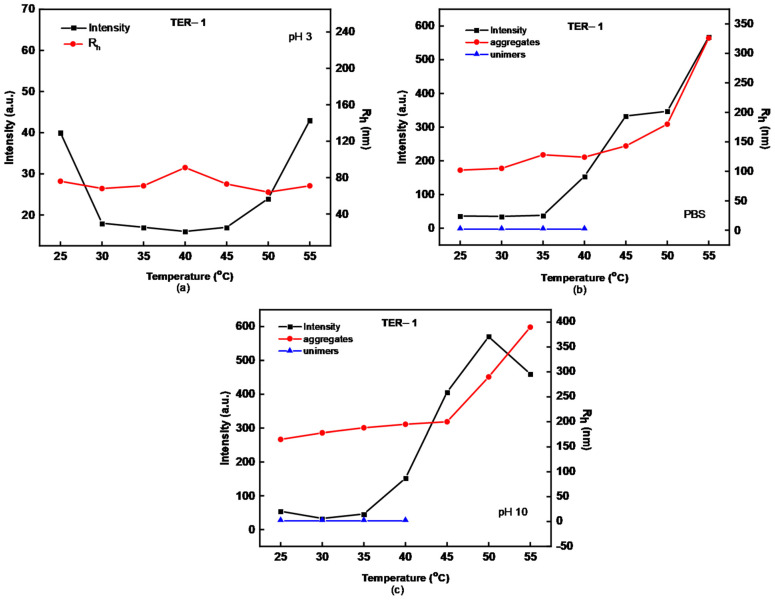
Scattering intensity (I) and the hydrodynamic radius (R_h_) as a function of temperature at (**a**) pH = 3, (**b**) PBS and (**c**) pH = 10 for TER-1 terpolymer.

Scattering intensity (I) and the hydrodynamic radius (R_h_) as a function of temperature are given in Figure 5 for the same polymer system.

The behavior of the system is important when switching from pH = 3 to PBS (pH = 7.4). In the PBS solution, two populations coexist, most probably free chains (unimers) and aggregates (multichain aggregates). Gradually, with increasing temperature, aggregates dominate, as was expected, since the hydrophobicity of the polymer chains increases. Correspondingly, the scattering intensity rises, which indicates a cumulative increase in the mass of the self-aggregating particles in solution. This is also related to the presence of salt in the solution in the case of PBS. The system shows a similar behavior at pH = 10, again with a predominance of aggregates and an increase in the scattering intensity, as temperature increases.

As shown in the plots (Figure 5), the temperature of 35 °C is the transition temperature for the system for solutions in PBS and at pH = 10. There is a sharp increase in the scattering intensity and, therefore, a rise in the mass of the aggregates formed with a parallel increase in R_h_. Similarly, the transition temperature was the same for the other systems. It is interesting to note the presence of small particles (unimers or small aggregates) up to 40 °C for PBS and pH = 10 solutions.

**Figure 6:**
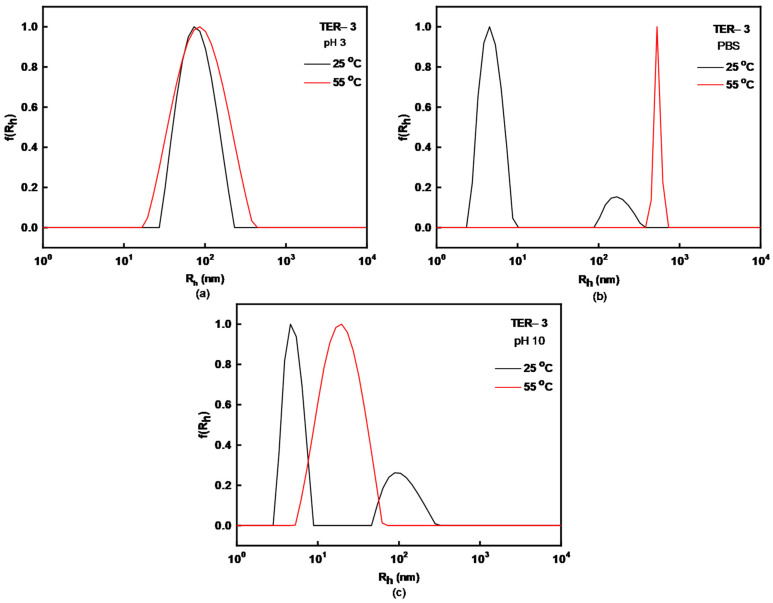
CONTIN size distributions for TER-3 terpolymer at (**a**) pH = 3, (**b**) PBS and (**c**) pH = 10 as a function of temperature.

**Figure 7:**
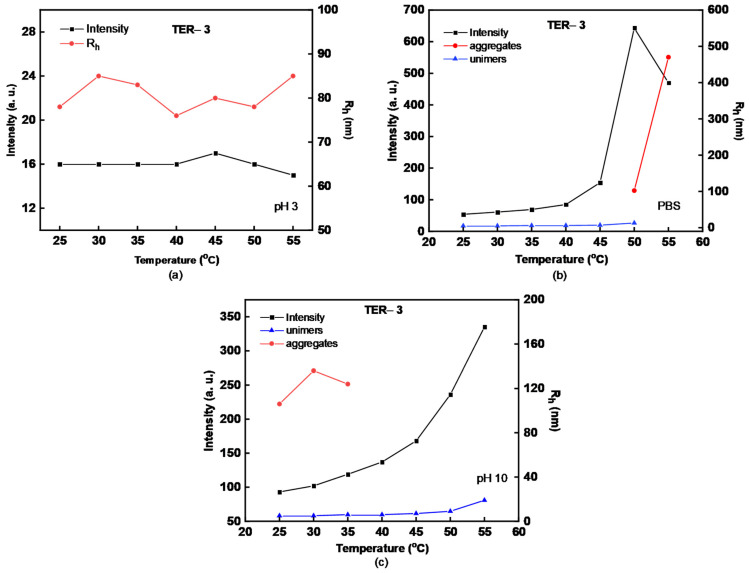
Scattering intensity (I) and the hydrodynamic radius (R_h_) as a function of temperature at (**a**) pH = 3, (**b**) PBS and (**c**) pH = 10 for TER-3 terpolymer.

In the case of TER-3 copolymer, as illustrated in Figure 6, there is a reduction in the DMAEMA content of almost half and an increase in both hydrophobic LMA and hydrophilic OEGMA contents (both non-responsive to temperature and pH). At pH = 3 there is one population, as before, with a relatively wide size distribution at both temperatures. In PBS solutions at 25 °C, two populations are revealed thatform nanoparticles with relatively narrow size distributions, with a predominance of larger particles, by increasing the temperature to 55 °C. At pH = 10 and 25 °C two populations are again present, while at 55 °C only one population prevails with intermediate dimensions. Representative plots for evaluation of scattering intensity and hydrodynamic radius, as a function of temperature and pH, are illustrated in Figure 7. The polymeric system shows stability at pH = 3, while in PBS solutions after 35 °C, there is an increase in the scattering intensity and an important predominance of aggregates, versus unimers. In addition, an increase in mass is evident. At pH = 10, in the absence of added salt, as the temperature increases, the large aggregates cease to prevail and give place to particles with smaller dimensions, while at the same time the intensity continues to increase. The reduction in the hydrodynamic radius may also be related to the shrinkage of the chains/aggregates, as temperature increases.

Next, electrophoretic light scattering measurements were performed, at pH = 3 and pH = 10. In an acidic environment PDMAEMA is protonated and is in cationic form and, therefore, the values ζ_p_ are expected to be positive. This coincides with the results presented in Table 2 for TER-1 and TER-3 copolymers. On the contrary, in an alkaline environment, where PDMAEMA is completely deprotonated, a reduction in ζ_p_ values is expected. More specifically, the resulting values were negative and far from zero. This is likely due to the –COOH end groups of the polymer chains originating from the CTA used, which due to the random arrangement are exposed to the surface of the particles and are deprotonated at pH = 10, displaying negatively charged groups –COO–. At the same time, there is a possibility that the negative charges at pH = 10 are due to adsorption of OH^−^ ions on the surface of the particles. It is worth noting the presence of unimers in this polymer system in a wider range of temperatures in PBS and pH = 10 solutions (Figure 7). For the rest of the polymeric systems, the results are presented in Table S3.

### 3.3. Effect of Ionic Strength on P(DMAEMA-co-LMA-co-OEGMA) Solution Assembly

**Figure 8:**
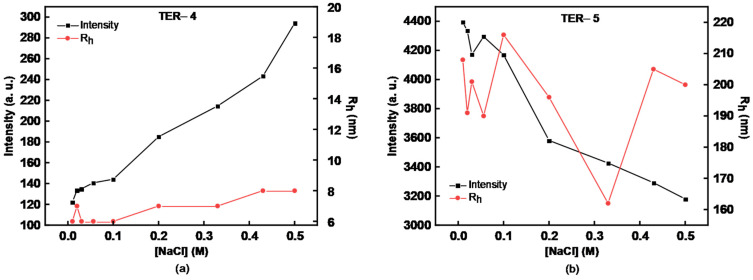
Ionic strength plots for (**a**) TER-4 and (**b**) TER-5 terpolymers (polymer concentration 10^−3^ g/mL, at pH = 3 and at a temperature of 25 °C).

Figure 8 represents the effect of ionic strength (IS) on the aggregation behavior of terpolymers with a different ratio of hydrophobic to hydrophilic components. For TER-4 copolymer, which is mainly hydrophobic, it is observed that there is an increase in the scattering intensity (I, proportional to the aggregate mass) at higher salt concentrations, while no significant changes are observed in terms of aggregate dimensions. Polymer–water interactions are not favored, on the contrary, there are mainly polymer–polymer interactions. However, a similar trend is not observed in TER-5 copolymer solutions, which has a higher DMAEMA content and a lower LMA content. Initially, the system shows a tendency for aggregation, as indicated by the increasing R_h_, but after the first five additions of salt (IS > 0.1 M NaCl) there is a decrease in scattering intensity that coincides with a sharp and temporary decrease in R_h_, leading to the reduction in the size of nanoparticles. With the further addition of salt, the system returns to its primary dimensions, probably due to polymer–polymer interactions that are enhanced because of salt saturation in the solution. PDMAEMA is a weak polybase and as the pH decreases, it is in cationic form, developing weak electrostatic interactions between the polymer chains, as the salt concentration increases. As a result, there is a “collapse” of the system, adeaggregation with a possible shrinking of the structures, which is also the reason for the reduction in size and scattering intensity.

The final R_h_ values, which appear to be relatively higher, are probably due to the solvent, which has penetrated inside the aggregates (swelling) due to the loosening of the structures in the presence of salt.

### 3.4. Curcumin Encapsulation in P(DMAEMA-co-LMA-co-OEGMA) Aggregates

In an attempt to study the ability of the terpolymers acting as drug carriers, curcumin was used as a model hydrophobic drug. The properties of the resulting systems were studied by dynamic light scattering, fluorescence spectroscopy and UV-Vis spectroscopy. The encapsulation was initially tested on all five terpolymers, as due to the different composition and statistical arrangement of the monomers, it is not possible to predict with certainty the behavior of the systems. The experimental procedure we followed is described in detail in Section 2.5. Two protocols were implemented to clarify which systems responded better to this role. The percent of curcumin encapsulated was calculated based on the weight composition of the terpolymers in LMA and the desired percentage of curcumin, attempted to be entrapped by weight. The samples were prepared with a maximum encapsulation level of 10% *w*/*w* and 20% *w*/*w* for all terpolymers in PBS, in respect to LMA content. The final concentration of the terpolymer solutions, after evaporation of the solvent (THF), was 10^−3^ g/mL.

UV-Vis spectroscopy was used as a first method in order to observe the absorption signal attributed to curcumin. However, successful encapsulation was visible to the naked eye, as the solutions were colored dark orange-red with the absence of any precipitation.

In general, it could be considered that by increasing the LMA content in the terpolymer there would be an enhancement in the entrapment level within the formed hydrophobic domains of the polymer aggregates. However, this is not a unique requirement, as the structure and stability of the system also depend significantly on the OEGMA monomer content and the overall structure of the mixed polymer–drug aggregates. Thus, it can be explained why the TER-4 copolymer did not show stability and, therefore, did not entrap curcumin sufficiently. The only measurement performed on this sample was by UV-Vis spectroscopy, which recorded almost zero absorption signal of curcumin in the solution.

Moreover, the 20% *w*/*w* curcumin systems with the protocol of organic solvent for TER-3 and TER-5 did not show stability and their study was not continued, as well as 20% *w*/*w* curcumin encapsulation for TER-5 with the thin-film protocol.

Below, are presented the results for TER-1 and TER-2/CUR mixed systems from the two protocols (Table 3). TER-1 and TER-2 are systems with almost the same composition but different molecular weights, with the TER-2 having about half the molecular weight of TER-1. So, the effects of polymer molecular weight may be distinguished.

The UV-Vis spectra were proportional for both protocols but gave different encapsulation levels in each case and, in particular, higher encapsulation efficiency (EE) in the thin-film protocol, where there was no effect on the thermo-responsive nature of the systems. Representative absorption plots are presented in Figures S1 and S2. The results for all the terpolymers are shown in Table S4.

DLS measurements for the stable systems followed. The solutions were measured at 25 °C on the first and tenth day after preparation to check their temporal stability. The results for all systems are presented in Table S5. In general, the picture given shows a relatively good stability as a function of time.

**Figure 9:**
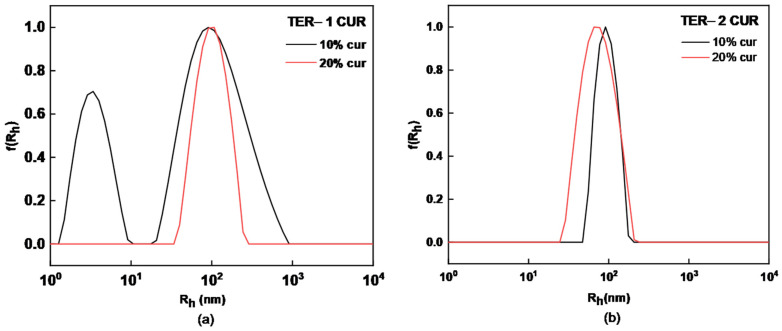
(**a**) TER-1 and (**b**) TER-2 terpolymer mixed aggregates with curcumin encapsulation 10% *w*/*w* and 20% *w*/*w* from THF protocol.

**Figure 10:**
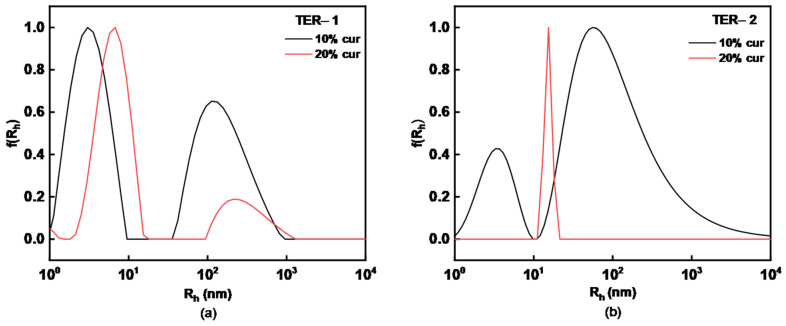
(**a**) TER-1 and (**b**) TER-2 terpolymer mixed aggregates with curcumin encapsulation 10% *w*/*w* and 20% *w*/*w* from thin-film protocol.

A single peak, with relatively narrow size distribution, shows that the polymeric aggregates that have entrapped the drug show colloidal stability, without being transformed into supra-aggregates. Generally, all the systems showed one population with the exception of TER-1 with 10% *w*/*w* curcumin, where two peaks were observed, while with an increase in the content of entrapped curcumin, the system eventually transforms to one population (Figure 9). Probably, the increase in the hydrophobic drug also helps in the better organization of the hydrophobic domains of the mixed polymer/drug aggregates and enhances their co-organization into nanoparticles. This can be confirmed in combination with the R_h_ results in the following table (Table 4). Comparatively, TER-2 copolymer is better organized, which, in both encapsulation rates, gave narrow peaks, low PDI values and relatively lower R_h_ values (Figure 9). These systems showed the same behavior in both protocols with better results when the percentage of encapsulation was 20% *w*/*w*, probably because the presence of curcumin actually helps in the construction of a more well-defined mixed nanostructure (Figure 10).

After the entrapment of CUR in the terpolymer aggregates, interaction of the mixed nanosystems with the proteins of bovine fetal serum was studied by DLS measurements. This test aimed at the possible biomedical utilization of the systems and the study of adverse interactions with blood components. FBS contains various proteins, mainly BSA (bovine serum albumin) at a content of about 2.5 mg/mL [27].DLS was performed for the determination of the characteristics and parameters, such as R_h_, PDI and scattering intensity (I), of the mixed nanoparticles in this blood simulating medium. The CUR-loaded nanoparticles into the FBS/PBS solution were prepared as described in Section 2.6.

**Figure 11:**
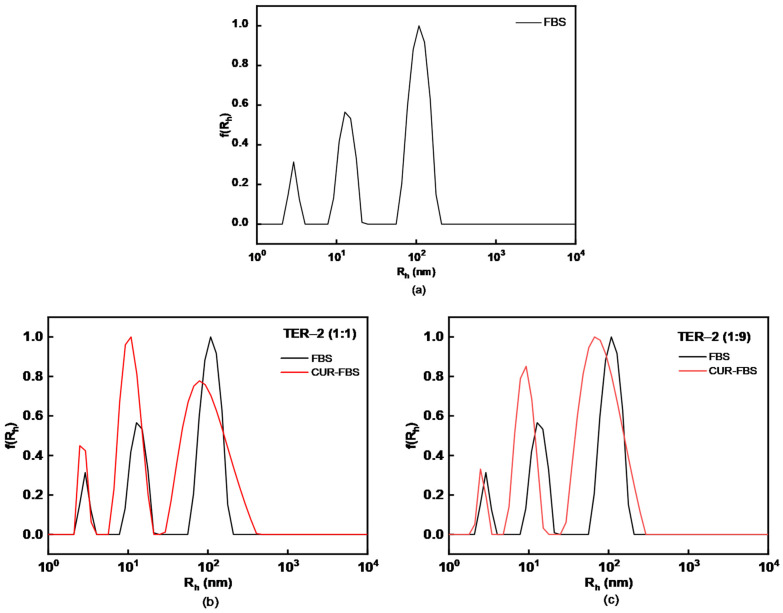
(**a**) Typical CONTIN size distribution of neat FBS, size distributions for (**b**) TER-2/CUR in FBS:PBS 1:1 and (**c**) TER-2/CUR in FBS:PBS 1:9.

FBS typically shows three peaks in the CONTIN analysis, as shown in Figure 11. From the DLS data illustrated in Figure 11, the CUR-loaded nanoparticles exhibit no further aggregation of the pre-existing mixed terpolymer-drug particles after mixing with FBS/PBS media. It is concluded that, in the FBS:PBS ratio 1:9, there is no interaction of the mixed nanostructures with the FBS components, and the loaded aggregates populations remain as they were. Respectively, three peaks are observed, indicating low interaction in the FBS:PBS ratio 1:1, with a lower intensity of the third peak due to the size overlap of the drug-loaded TER-2 system with proteins. Therefore, CUR-loaded terpolymer aggregates exhibit substantial stability in the presence of serum proteins.

Based on extensive literature that exists regarding the optical properties of curcumin, it has been proven that curcumin provides strong intrinsic fluorescence, a property that can be useful for bio-imaging applications [28]. Therefore, fluorescence studies of CUR-loaded terpolymers were performed under physiological conditions in PBS solutions. It is known from literature that the solubility of curcumin is very limited in water (4.2 μg/mL) [29,30]. CUR solubility is significantly improved by encapsulation in the hydrophobic domains of the terpolymer aggregates. After CUR entrapment, the loaded polymeric aggregates exhibit significant fluorescence.

**Figure 12:**
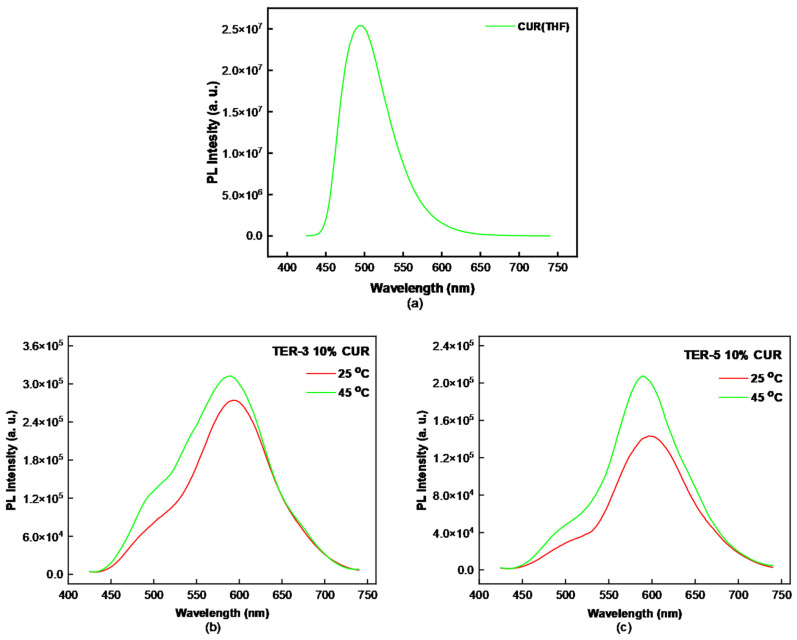
(**a**) Fluorescence of curcumin in THF (**b**) TER-3/CUR (**c**) TER-5/CUR mixed aggregates fluorescence signal as a function of temperature.

According to the literature, curcumin in THF solutions gives a fluorescent signal at 500–520 nm. The systems being studied, however, gave a curcumin signal at higher values, at 580–600 nm (Figure 12) [31].

Based on other studies performed using curcumin as a drug and fluorescent tracer, it is concluded that values close to 570 nm (and higher) are probably due to aggregation of curcumin in the polymeric aggregates [31]. Specifically, curcumin molecules aggregate in the hydrophobic domains, resulting in dimerization, along with their interaction with polymeric chains. This aggregation leads to a reduction in the π-π* gap between curcumin molecular orbitals.

At the same time, due to DMAEMA dimethylamino groups, there is high possibility for an acid–base interaction between DMAEMA –N(CH_3_)_2_ groups and curcumin phenolic hydroxyls. This would lead to the ionization of the DMAEMA dimethylamino groups and could give further interactions with the system, beyond the formation of H-bonds within the hydrophobic core [31,32]. So, one scenario that would explain the spectra taken in our systems is that curcumin is not exclusively trapped in the hydrophobic domains of the terpolymer aggregates, surrounded by the rest of the polymer, but also scattered within the polymer matrix of the statistical terpolymer aggregates due to interaction with the more hydrophilic DMAEMA segments. As a result, curcumin behaves more like aggregated/complexed curcumin and not as solubilized curcumin molecules into the hydrophobic domains nanoparticles, showing absorption at higher wavelengths. One should have in mind that hydrophobic domains in the present terpolymer aggregates may not be large enough, due to the statistical nature of the terpolymer chain with LMA segments being randomly distributed among hydrophilic DMAEMA and OEGMA segments.

Finally, it is also worth pointing out the behavior of the fluorescent TER/CUR mixed systems as a function of temperature. As temperature increases from 25 °C to 45 °C, there is an increase in fluorescent intensity of the solutions, as shown in Figure 12. This trend was observed in all systems that curcumin was entrapped, by both protocols and at both encapsulation levels, confirming once again the mixed system response to temperature changes. As has already been mentioned, the rise in temperature leads to enhance the hydrophobic character of the DMAEMA and consequently to the reorganization of the polymer/drug nanoparticles as a whole, which, in turn, affects curcumin aggregation state and interactions in the mixed aggregates and its fluorescence properties. Such nanosystems may find application in the detection of temperature changes within biological tissues in vivo after certain optimization. The present observations give good ground as preliminary proof of concept studies.

## 4. Conclusions

The synthesis of five amphiphilic statistical terpolymers of the type P(DMAEMA-co-LMA-co-OEGMA) having different compositions in each monomer was achieved via RAFT polymerization. The success of the synthesis was confirmed by the molecular characterization, using chromatographic and spectroscopic techniques. Studies on aqueous solutions of the dual-responsive nanosystems using a gamut of physicochemical techniques (including DLS, ELS and FS) revealed the response of the P(DMAEMA-co-LMA-co-OEGMA) terpolymers to pH, temperature and ionic strength changes.

It was also shown that the terpolymers can be used as vehicles for the encapsulation of curcumin. Each terpolymer system, depending on its composition, revealed a different behavior and performed differently in terms of drug encapsulation. The architecture of the systems also contributed to this, as there are no distinct and large domains into which curcumin could be entrapped. Their colloidal stability was maintained for more than 10 days after CUR encapsulation. Studies with FBS-containing solutions indicate that the CUR-polymer systems have no tendency to interact with serum proteins, probably due to the presence of OEGMA units. Additionally, the CUR-loaded P(DMAEMA-co-LMA-co-OEGMA) aggregates displayed considerable fluorescence, which was also found to be sensitive to solution temperature changes, thus, showing the potential to be utilized in bio-imaging applications in vivo. Based on the results at hand, these amphiphilic statistical P(DMAEMA-co-LMA-co-OEGMA) terpolymers show multifaceted stimuli-responsive self-assembly in aqueous media, forming aggregates that could be promising drug vehicles, both for drug delivery and bio-imaging applications.

## Figures and Tables

**Table 1:** Molecular characteristics of the five P(DMAEMA-co-LMA-co-OEGMA) terpolymers.

Sample	M_W_ (×10^4^) (g/mol) ^a^	M_W_/M_n_^a^	%wt DMAEMA ^b^	%wt LMA ^b^	%wt OEGMA ^b^	% Yield
TER-1	1.8	1.09	66	10	24	66.7
TER-2	1.1	1.16	62	11	27	70.5
TER-3	1.72	1.1	35	29	36	53.3
TER-4	1.67	1.09	20	48	32	92.4
TER-5	1.4	1.1	49	27	24	76.7

^a^: Determined by SEC. ^b^: Determined by ^1^H-NMR.

**Table 2:** Structural characteristics of TER-1 and TER-3 terpolymers in aqueous media.

Sample	T (°C)	pH	Intensity	R_h_ (nm)	PDI	ζ_p_ (mv)
TER-1	25 °C	pH 3	40	76	0.6	26.5
		PBS	36	3/102	0.6	–
		pH 10	54	3/165	0.6	−35.5
	55 °C	pH 3	43	71	0.5	–
		PBS	567	326	0.3	–
		pH 10	460	390	0.2	–
TER-3	25 °C	pH 3	16	78	0.7	5.0
		PBS	54	5/173	0.4	–
		pH 10	93	5/106	0.4	−18.0
	55 °C	pH 3	15	85	0.6	–
		PBS	470	532	3	–
		pH 10	335	19	0.2	–

**Table 3:** Curcumin encapsulation efficiency for P(DMAEMA-co-LMA-co-OEGMA) copolymers.

Sample *	Protocol	CUR Used (mg)	Max Encapsulation (% *w*/*w*)	% Encapsulation Efficiency (EE)	% Drug Loading (DL)
TER-1	THF	0.1	10	55.2	0.55
		0.2	20	49.7	0.99
	Thin film	0.1	10	83.4	0.83
		0.2	20	92.2	1.8
TER-2	THF	0.1	10	68.0	0.75
		0.2	20	47.4	1.04
	Thin film	0.1	10	70.9	0.78
		0.2	20	95.7	2.11

* 10 mg of each terpolymer were used for each preparation.

**Table 4:** DLS results before and after curcumin encapsulation in P(DMAEMA-co-LMA-co-OEGMA) aggregates.

Sample *	Protocol	R_h_ (nm) without CUR	PDI without CUR	% CUR	R_h_ (nm) with CUR	PDI with CUR
TER-1	THF	3/102	0.6	10	3/112	0.5
				20	99	0.5
	Thin film	3/100	0.5	10	3/149	0.4
				20	6/288	0.5
TER-2	THF	3/108	0.5	10	93	0.3
				20	73	0.2
	Thin film	72	0.2	10	3/108	0.6
				20	15.2	0.009

* 10 mg of each terpolymer were used for each preparation.

## Data Availability

The data presented in this study are available on request from the corresponding author.

## Supplementary Materials

The following supporting information can be downloaded at: https://www.mdpi.com/article/10.3390/nano12213791/s1, Table S1: Quantities of reagents used for the synthesis of P(DMAEMA-co-LMA-co-OEGMA) terpolymers; Table S2: CAC values and Ι_1_/Ι_3_ ratios for the statistical P(DMAEMA-co-LMA-co-OEGMA) terpolymer aggregates at 25 °C and 55 °C in aqueous solutions; Table S3: Structural characteristics of the P(DMAEMA-co-LMA-co-OEGMA) copolymer aggregates in aqueous media; Figure S1: UV-Vis spectra for TER-1 (CUR) (left) and TER-2 (CUR) (right) mixed aggregates aqueous solutions from the THF protocol; Figure S2: UV-Vis spectra for TER-1 (CUR) (left) and TER-2 (CUR) (right) mixed aggregates in aqueous media from the Thin Film protocol; Table S4: Results on curcumin encapsulation efficiency for P(DMAEMA-co-LMA-co-OEGMA) copolymers for both encapsulation protocols; Table S5: DLS results from aqueous solutions the 1stand the 10th day from the preparation of the terpolymer—curcumin mixed aggregates.
