# Supplementary material for: Dual-Responsive Amphiphilic P(DMAEMA-co-LMA-co-OEGMA) Terpolymer Nano-Assemblies in Aqueous Media
Source: Nanomaterials (Basel). 2022 Oct 27;12(21):3791. doi: 10.3390/nano12213791 (PMC9659099; doi:10.3390/nano12213791)
Supplement: Supplementary file 1 [file nanomaterials-12-03791-s001.zip › nanomaterials-1946964-supplementary.pdf]

Supplementary Materials

Dual-Responsive Amphiphilic P(DMAEMA-co-LMA-co-OEGMA) Terpolymer Nano-Assemblies in Aqueous Media

Maria Tomara, Dimitrios Selianitis and Stergios Pispas *

Theoretical and Physical Chemistry Institute, National Hellenic Research Foundation, 48 Vassileos Constantinou Avenue, 11635 Athens, Greece

* Correspondence: pispas@eie.gr; Tel.: +30-210-727-3824

Table S1. Quantities of reagents used for the synthesis of P(DMAEMA-co-LMA-co-OEGMA) terpolymers.

Sample	DMAEMA (mL)	LMA (mL)	OEGMA (mL)	CTA (mg)	AIBN (mg)
TER-1	1.29	0.23	0.58	28	3.3
TER-2	1.29	0.23	0.58	56	6.6
TER-3	0.67	0.72	0.815	28	3.3
TER-4	0.45	1.21	0.61	28	3.3
TER-5	0.9	0.48	0.82	28	3.3

Table S2. CAC values and I_1/I_3 ratios for the statistical P(DMAEMA-co-LMA-co-OEGMA) terpolymer aggregates at 25 °C and 55 °C in aqueous solutions.

Sample	pH	I_1/I_3		CAC
		25 °C	55 °C	
TER-1	pH 3	1.39	1.41	–
	PBS	1.17	1.24	9.24×10^{-6}
	pH 10	1.15	1.21	1.04×10^{-5}
TER-2	pH 3	1.38	1.35	–
	PBS	1.22	1.28	1.07×10^{-5}
	pH 10	1.20	1.23	5.56×10^{-5}
TER-3	pH 3	1.17	1.19	–
	PBS	1.01	0.94	4.46×10^{-6}
	pH 10	1.00	1.00	4.26×10^{-6}
TER-4	pH 3	1.05	1.03	–
	PBS	0.99	0.92	5.97×10^{-7}
	pH 10	0.98	0.99	1.10×10^{-6}
TER-5	pH 3	1.23	1.20	–
	PBS	1.00	1.03	1.17×10^{-5}
	pH 10	1.01	1.00	7.53×10^{-6}

From the table it is obvious that TER-4 copolymer, with the higher LMA content, has the lowest CAC, as well as I_1/I_3 ratio (more hydrophobic domains). However, the DMAEMA content is also important since this is the responsive monomeric unit. The results clearly show the change, which is proportional to the composition of each copolymer in terms of the responsive DMAEMA content, and in combination with the content of hydrophobic LMA monomer.

Table S3. Structural characteristics of the P(DMAEMA-co-LMA-co-OEGMA) copolymer aggregates in aqueous media.

Sample	T (°C)	pH	Intensity (KHz/s)	R_h (nm)	PDI	ζ_p (mV)
TER-1	25 °C	pH 3	40	76	0.6	26.5
		PBS	36	3 / 102	0.6	–
		pH 10	54	3 / 165	0.6	−35.5
	55 °C	pH 3	43	71	0.5	–
		PBS	567	326	0.3	–
		pH 10	460	390	0.2	–
TER-2	25 °C	pH 3	22	113	0.6	12.5
		PBS	37	3 / 108	0.5	–
		pH10	21	3	0.6	−30.0
	55 °C	pH 3	568	112	0.2	–
		PBS	6340	284	9	–
		pH 10	3940	423	0.07	–
TER-3	25 °C	pH 3	16	78	0.7	5.0
		PBS	54	5 / 173	0.4	–
		pH 10	93	5 / 106	0.4	−18.0
	55 °C	pH 3	15	85	0.6	–
		PBS	470	532	3	–
		pH 10	335	19	0.2	–
TER-4	25 °C	pH 3	67	5	0.4	7.0
		PBS	5360	98	0.4	–
		pH 10	1016	11	0.2	−29.4
	55 °C	pH 3	60	4	0.4	–
		PBS	41,500	502	0.3	–
		pH 10	1661	12 / 99	0.3	–
TER-5	25 °C	pH 3	612	131	0.2	74.2
		PBS	107	5 / 156	0.5	–
		pH 10	94	6	0.4	−32.0
	55 °C	pH 3	231	139	0.2	–
		PBS	12,580	124	21	–
		pH 10	911	13	0.11	–

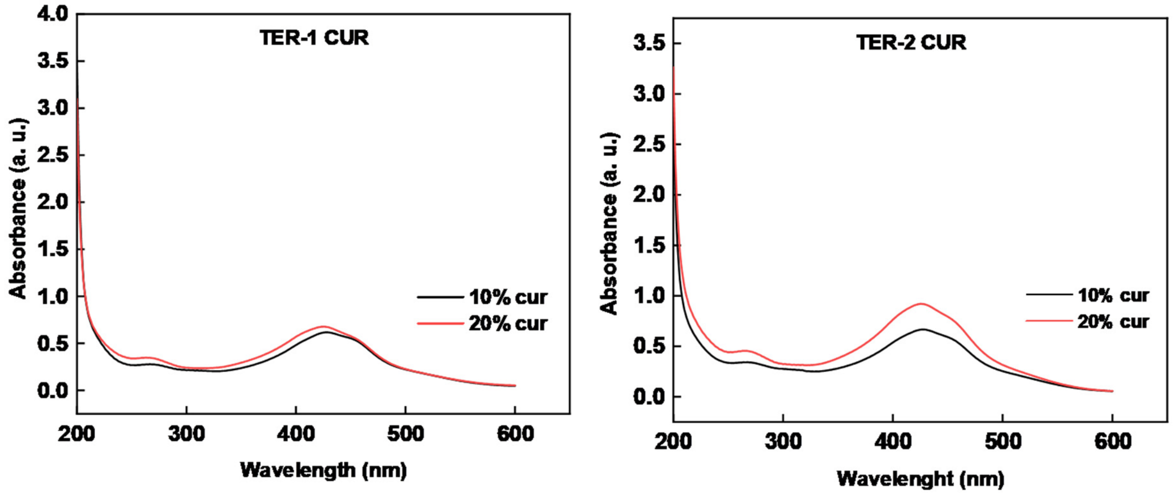

Figure S1. UV-Vis spectra for TER-1 (CUR) (**left**) and TER-2 (CUR) (**right**) mixed aggregates aqueous solutions from the THF protocol.

From the UV-Vis spectra for TER-1 and TER-2 copolymer CUR mixed aggregates it is observed that an increase of the curcumin content does not lead to a corresponding increase in the absorption signal. In TER-2 terpolymer, however, there is a larger variation (Figure S1).

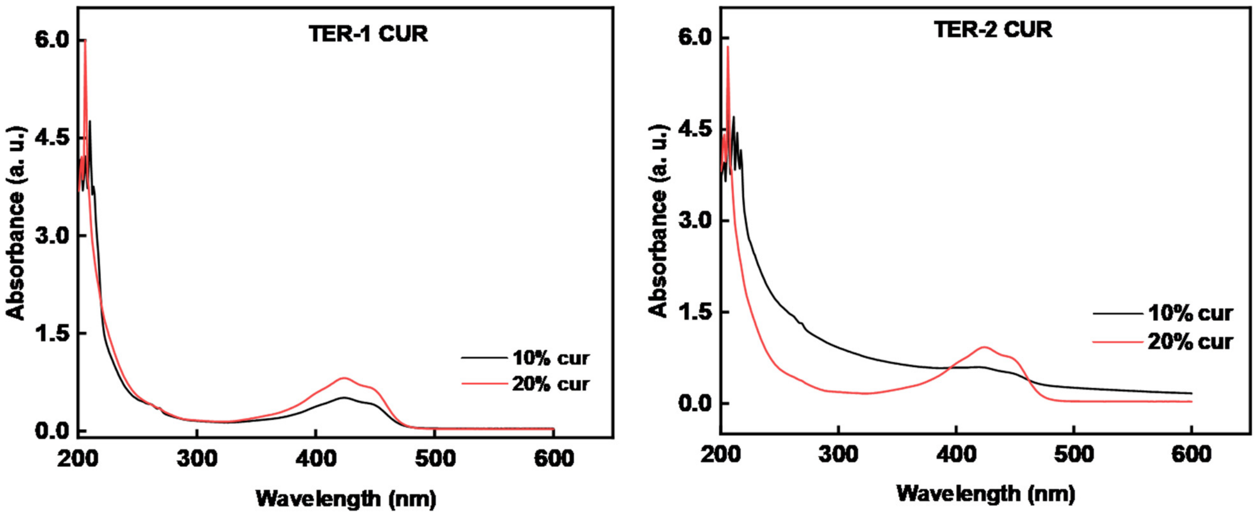

Figure S2. UV-Vis spectra for TER-1 (CUR) (**left**) and TER-2 (CUR) (**right**) mixed aggregates in aqueous media from the thin film protocol.

Table S4. Results on curcumin encapsulation efficiency for P(DMAEMA-co-LMA-co-OEGMA) copolymers for both encapsulation protocols.

Sample*	Protocol	Max CUR used (mg)	Max percentage of encapsulation (% *w*/*w*)	% encapsulation efficiency (EE)	% drug loading (DL)
TER – 1	THF	0.1	10	55.2	0.55
		0.2	20	49.7	0.99
	Thin film	0.1	10	83.4	0.83
		0.2	20	92.2	1.8
TER – 2	THF	0.11	10	68.0	0.75
		0.22	20	47.4	1.04
	Thin film	0.11	10	70.9	0.78
		0.22	20	95.7	2.11
TER – 3	THF	0.29	10	69.9	2.03
	Thin film	0.29	10	71.7	2.08
		0.58	20	12.5	0.72
TER – 5	THF	0.27	10	72.0	1.95
	Thin film	0.27	10	78.2	2.11

* 10 mg of each terpolymer were used for each preparation.

Table S5. DLS results from aqueous solutions the 1st and the 10th day from the preparation of the terpolymer – curcumin mixed aggregates.

Sample	Protocol	R_h (nm) withoutCUR	R_h (nm) with CUR (1st day)	PDI (1st day)	R_h (nm) with CUR (10th day)	PDI (10th day)
TER – 1	THF	3/102	3/112	0.5	4/104	0.5
			99	0.5	4/95	0.6
TER – 2	THF	3/108	93	0.3	104	0.5
			73	0.2	81	0.3
TER – 3	THF	3/157	152	0.3	182	0.3
TER – 5	THF	5/156	156	0.2	173	0.2
